# ZnAl-SO_4_ Layered Double Hydroxide and Allophane for Cr(VI), Cu(II) and Fe(III) Adsorption in Wastewater: Structure Comparison and Synergistic Effects

**DOI:** 10.3390/ma15196887

**Published:** 2022-10-04

**Authors:** Anna Maria Cardinale, Cristina Carbone, Marco Fortunato, Bruno Fabiano, Andrea Pietro Reverberi

**Affiliations:** 1 DCCI, Department of Chemistry and Industrial Chemistry, Università degli Studi di Genova, Via Dodecaneso 31, 16146 Genova, Italy; 2 Dipartimento per lo Studio del Territorio, dell’Ambiente e della Vita, Università di Genova, Corso Europa 26, 16146 Genova, Italy; 3 DICCA, Department of Civil, Chemical and Environmental Engineering, Polytechnic School, Università degli Studi di Genova, Via Opera Pia 15, 16145 Genova, Italy

**Keywords:** allophane, layered double hydroxides, wastewater, adsorption, heavy metals

## Abstract

Owing to their structure, layered double hydroxides (LDHs) and allophane are nowadays considered as promising materials for application in different fields. The goal of this work is to compare the efficacy of allophane and ZnAl-SO_4_ LDH to remove, by adsorption, some cationic and anionic pollutants from industrial wastewater. Both compounds were synthesized via the co-precipitation route (direct method) followed by hydrothermal treatment, obtaining nanoscopic crystallites with a partially disordered turbostratic (ZnAl-SO_4_ LDH) or amorphous (allophane) structure. The characterization of the obtained compounds was performed by means of powder x-ray diffraction (PXRD), thermal gravimetry analysis (TGA), field emission scanning electron microscopy analysis (FESEM), and Fourier-transform infrared spectroscopy (FT-IR). The sorbents were tested using wastewater produced by a real metalworking plant and containing ionic species such as Cu(II), Fe(III) and Cr(VI), whose concentration was measured by means of inductively coupled plasma-optical emission spectrometry (ICP-OES). This investigation represents an alternative procedure with respect to standard protocols based on customarily made and artificially lab-produced wastewaters. Both sorbents and their combination proved to be efficient in Cr(VI) removal, irrespective of the presence of cations like Cu(II) and Fe(III). A synergistic effect was detected for Cu(II) adsorption in a mixed allophane/LDH sorbent, leading to a Cu(II) removal rate of 89.5%.

## 1. Introduction

The development of industrial and technological society dramatically increased the number of metals released into the environment by anthropogenic activity. In the last decades, much effort has been devoted to finding economic and eco-friendly routines (the so-called “green technologies”) to prevent heavy metal contamination in surrounding soils and water as an effect of industrial wastewater release in the environment [1].

To this purpose, one of the most widespread techniques for the abatement of polluting agents in water relies upon adsorption processes, whose substrates have been object of intense investigations in the last decades [2]. As a first rough classification, adsorbing materials can be divided into organic [3], inorganic [4] and hybrid substrates [5].

The former are constantly growing in number and varieties, in compliance with the recent sustainability protocols, where the minimization of environmental risk factors together with an implementation of recycling strategies are the main targets of modern manufacturing. Anderson et al. [6] highlighted the importance of biodegradable materials as a promising class of sorbents, opening future scenarios for the abatement of polluting cations in wastewater. To this purpose, many different materials of vegetal origin have been proposed in the literature [7], including fruit peels [8], pine bark [9], rice husk [10] and many other plant derivatives, in natural or modified forms [11], which may represent a challenging strategy of combining pollution control with a recovery of an otherwise useless or even noxious by-products.

In a slightly different, though related, context pertaining to organic sorbents of non-vegetal origin, many researchers have focused on the use of biomacromolecules of animal origin, such as chitosan [12]. Chitosan is a very versatile biopolymer which lends itself to a multiplicity of functions, [13] leading to very attractive utilizations not only limited to pollution control. Likewise, polymers of artificial origin, relying upon thermoplastics further engineered in nanofibers, proved to be advantageous in the abatement of Pb (II) ions in aqueous mediums [14]. Needless to say, nanotechnology may be strategical in many cases, owing to its capability of revamping the uses of old or exhausted materials, possibly applying cleaner and inherently safer approaches [15] to gain new and often unexpected surface properties important in adsorption phenomena.

Inorganic matrices, historically representing a cornerstone as adsorbing materials, do not seem to have had a development comparable to that of organic substrates in recent times. However, adsorption by minerals and/or by mineral-derived materials remains a widely applied technique owing to its ease of use and low cost. They can be of natural or synthetic origin and, in some cases, may offer a constancy of physicochemical properties unsurpassed by more recent green organic sorbents of natural origin [16]. The adsorptive materials include alumina, zeolites, clays [17] and hydroxyapatite [18], and novel adsorbents such as nanomaterials, magnetic materials [19] and hybrid organic-inorganic matrices, such as metal-organic frameworks (MOF) [20]. Titanosilicates are a wide class of natural and artificial sorbents, originally used as molecular sieves, having extended uses in both cation adsorption and in cation exchange [21]. As pointed out by Oleksiienko et al. [22], they proved to be highly selective over a large pH range, and offered the unusual peculiarity of a growing sorption ability for a decreasing structural order of the crystalline lattice.

Layered double hydroxides (LDHs) and allophane [23] are attracting increasing attention due to their simple and cost-effective synthesis routines and tenability of their properties.

LDHs are layered materials with brucite-type layers where a trivalent cation (M^3+^) partially substitutes a divalent cation (M^2+^), generating a net positive charge balanced by an anionic species (A^z−^) in the interlayer, giving a general formula M^2+^_1−x_M^3+^_x_(A^z−^)_x/z_(OH)_2_·nH_2_O [24]. LDH are also called “anionic clays” owing to their characteristics as anionic exchangers [25]. The particular feature derived from their structure [26] makes these compounds promising materials for a wide range of technological applications in ion exchange/adsorption, pharmaceutics [27], electrochemistry [28], catalysis and photocatalysis. In the latter field of research, ZnAl LDH nanosheets are in the hotspot for their specific ability to fix atmospheric nitrogen with the formation of ammonia by UV light under mild conditions [29]. LDH properties in capturing ions have been successfully exploited in environmental decontamination [30] and in civil engineering [31], as LDH may prolong the life of reinforced concrete structures by entrapping Cl^−^ ions, thus damping the corrosion of steel.

Allophane or allophanic materials are hydrous aluminosilicates Al_2_O_3_⋅(SiO_2_)_1,3-2_⋅((H_2_O))_2,5-3_ with a variable Al/Si ratio, which lead to differences in structure and chemical composition reflecting on their features [32]. Allophane spheres are composed by a gibbsite sheet outer sphere (octahedral Al) and an imogolite-like (tetrahedral Si) inner sphere. The primary spherules contain some defects or open pores of about 3.5 Å in diameter along the walls [33]. The spherules form clusters, or small aggregates, of 8–10 nm size, which, in turn, stick together to form larger, up to 60–100 nm aggregates. This sorbent has been proposed in different formulations, namely in its natural, activated [34] and even organic-composite form, where it has been tested as a substrate for micro-organisms in aerobic batch reactors [35]. An important characteristic of allophane, deriving from its structure, is its high cation exchange capability (CEC), but also anions are adsorbed by this material [36].

The aim of this work is to test the ability of synthetic ZnAl-SO_4_ LDH and allophane to adsorb heavy metals from wastewater, where the metals investigated are Cr(VI) anion, Fe(III) and Cu(II) cations. Such polluting ions have been intentionally chosen, owing to their recurrent presence, not only limited to metalworking plants or machining processes [37], but likewise extended to a wide variety of effluents produced by many other chemical processes typical of tanneries, the galvanic industry and hydrometallurgical treatments. The paper is divided as follows. Firstly, the methods adopted to synthesize the aforementioned sorbents are described, together with the relevant characterization techniques aiming at investigating the structure of the relevant solid substrates. Afterwards, the physicochemical properties of the substrates are described and compared with analogous results taken from literature. Furthermore, some sorption tests at equilibrium have been carried out in order to ascertain the best yields and selectivity towards different ions contained in wastewater produced by a metalworking plant. Finally, the conclusions are drawn and a direction for future applications of the present study is traced.

## 2. Materials and Methods

### 2.1. Reagents for Sorbent Preparation

Zinc sulphate (ZnSO_4_·7H_2_O, 99%, VWR Chemicals, Leuven, Belgium), anhydrous aluminium sulphate (Al_2_(SO_4_)_3_, 99%, VWR Chemicals, Leuven, Belgium), anhydrous sodium hydroxide (NaOH, ≥98%, Sigma-Aldrich, Milano, Italy), sodium orthosilicate (Na_4_SiO_4_, 99%, Thermo Fisher, Kandel, Germany) and aluminium chloride (AlCl_3_·6H_2_O, 99%, Merck, Milano, Italy) were employed as supplied. Deionized water was used in all experimental samples and it was purged with argon to remove the presence of CO_2_ traces incidentally present when required.

### 2.2. LDH Synthesis

Both compounds, LDH and allophane, were synthesized through co-precipitation (direct method) followed by hydrothermal treatment in order to obtain nanoscopic crystallites.

The ZnAl-SO_4_ LDH was prepared with the atomic ratio M^2+^/M^3+^ = 3 according to the protocol proposed by Bîrjega et al. [38], who synthesized rare-earth modified mesoporous hydrotalcites as selective catalysts in cyanoethylation of ethanol with acrylonitrile.

The whole procedure was carried out at room temperature and under inert atmosphere so as to avoid the presence of carbonate anion in the interlayer.

45 mmol of ZnSO_4_∙7H_2_O and 7.5 mmol of Al_2_(SO_4_)_3_ were solubilized in a beaker containing 150 mL of deionized water.

Separately, 200 mL of deionized water was inserted into a three-necked flask equipped with a magnetic stirrer and an electrode for pH measurement. The pH value was monitored by means of a pH-meter pH/ORP/ISE Single Channel Benchtop Meter-HI3221 (HANNA Instruments, Woonsocket, RI, USA). The solution containing the mixed sulphates was added dropwise into the flask, keeping the pH constantly fixed at 8 ± 0.5 by continuous corrections with NaOH. The sodium hydroxide here employed was prepared in different concentrations from 0.5 M to 2 M to be used depending on the extent of the necessary correction.

During the dripping of the mixed sulphate solution, the solid precursor of the LDH precipitates. Afterwards, the solid and the solution were transferred completely from the reaction flask into a dark glass bottle and the suspension was aged in a stove at 50 °C for a week. During the process of digestion of the precipitate, the solid LDH phase acquired a crystalline structure. Subsequently, the precipitate containing crystalline LDH was separated by filtration, washed with water and dried in a static oven at 50 °C for 24 h.

### 2.3. Allophane Synthesis

In this case [39], two aqueous solutions of Na_4_SiO_4_ 0.1 M and AlCl_3_∙6H_2_O 0.1 M, respectively, were mixed in a proper amount and the mixture was kept under magnetic stirring in open air for 1 h. The gelatinous solid obtained according to reaction (1) is the allophane precursor.

Na_4_SiO_4_ + 4/3AlCl_3_ → SiO_2_∙ 2/3Al_2_O_3_ + 4NaCl (1)

The solid was separated by settling, filtered and repeatedly washed up to the complete elimination of sodium chloride. After this procedure, the precursor was transferred with water in a Teflon vessel autoclave and heated in an oil bath at 100 °C for 48 h. During heating, a transformation occured, leading to a refinement of the microstructure, reducing the size of the primary particles down to a nanometric scale, thus increasing the contact surface available for adsorption processes. After the heating phase, the as-formed allophane was vacuum filtered and dried at 50 °C in a static oven for 24 h.

### 2.4. Characterization Techniques

As previously mentioned, the structural properties of the synthesized samples were investigated by means of thermal gravimetric analysis (TGA), x-ray analysis on powders (PXRD), field emission scanning electron microscopy (FESEM) and Fourier-transform infrared spectroscopy (FT-IR). The concentration of polluting cations in the solution embedding the suspended sorbent was measured by inductively coupled plasma-optical emission spectrometry (ICP-OES).

TG/DTA analysis was performed using a H/LABSYS EVO-1A SETARAM apparatus (Setaram, Caluire, France); about 20 mg of sample was placed in an open alumina crucible and heated under argon flux at 30 mL/min from 30 °C to 1250 °C at 5 °C/min.

PXRD was realized with a vertical diffractometer X’Pert MPD (Philips, Almelo, The Netherlands) equipped with a Cu tube (CuKα; wavelenght *λ*: 1.54050 Å). The samples were ground in an agate mortar and the patterns were collected according to an angle gap 2θ between 10° and 100°, applying a scanning rate of 0.001° with a measuring time of 50 s/step. The indexing of the obtained diffraction peaks was performed by comparison with literature or calculated data, using the software Powder Cell [40], whereas the lattice parameters of the LDHs were calculated using the software LATCON [41]. The powder diffraction pattern analysis was carried out by adopting the package FullProf Suite [42], relying upon the Rietveld refining technique, which proved to be an useful tool in nanopowders characterization [43].

A FESEM analysis was performed to reveal the samples’ morphology. After adhesion on a conductive resin support, the specimens were analyzed applying an acceleration voltage of 5 kV for 50 s, and a cobalt standard was used for calibration.

To check the structure of the two compounds and for the LDH, to exclude the presence of molecular species different from the sulphate in the interlayer (e.g., CO_2_ from atmosphere), FTIR spectroscopy was performed by means of a Spectrum 65 FT-IR Spectrometer (PerkinElmer, Waltham, MA, USA) equipped with a KBr beamsplitter and a DTGS detector using an ATR accessory with a diamond crystal. All the spectra were recorded from 4000 cm^−1^ to 600 cm^−1^.

The ICP-OES measurements were performed using an axially viewed Varian (Springvale, Australia) Vista PRO. The sample introduction system consisted of a glass concentric K-style pneumatic nebulizer (Varian) jointed to a glass cyclonic spray chamber (Varian).

## 3. Results and Discussion

The solid substrates, obtained according to the experimental techniques described in Section 2.2 and Section 2.3, were investigated in their physicochemical properties related to structure and composition. In a second phase, the sorbents were tested in order to check their performances in wastewater decontamination.

### 3.1. Structural (PXRD, IR and TG) and Morphological (FESEM) Analysis

**Figure 1:**
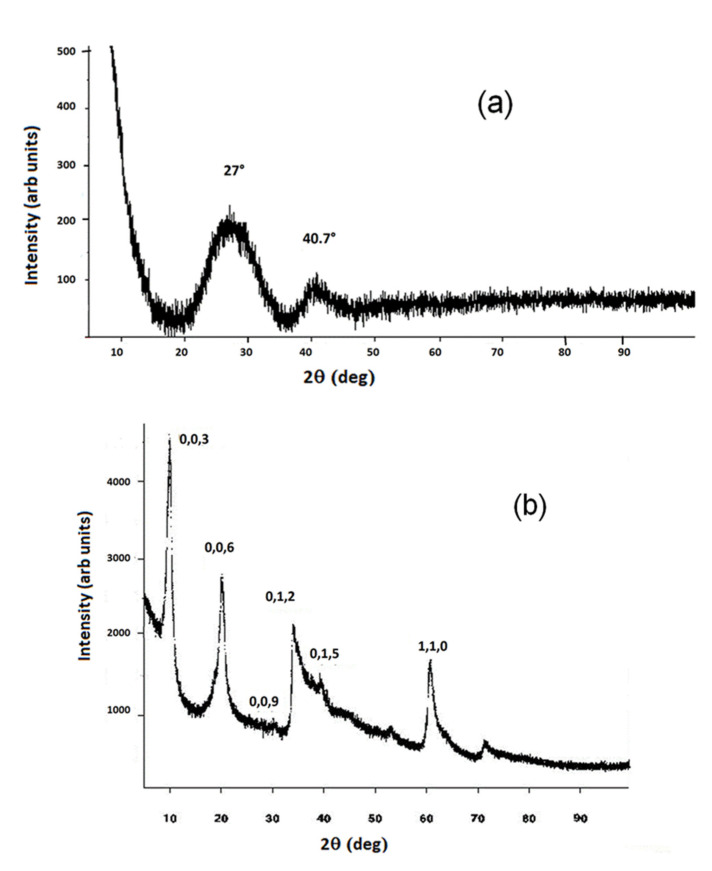
XRD patterns pertaining to allophane (**a**) and to ZnAl-SO_4_ LDH (**b**).

The PXRD pattern in Figure 1a confirms the typical allophane amorphous structure [44], with a characteristic broad peak at 2θ about 27.0° and 40.7° (CuKα), typical of a short-range order aluminosilicate.

The ZnAl-SO_4_ XRD pattern in Figure 1b (with the typical turbostratic structure) shows the main typical symmetric reflections at 003 = 10.04°, 006 = 20.23°, 009 = 30.13°, 110 = 60.71° and asymmetric 012 = 34.86° and 015 = 38.09° reflections. The crystal structure was determined and the cell parameters were calculated with the following results: a = 0.3044(6) nm, c = 2.6549(9) nm, Vcell = 0.213(1) nm^3^. The brucite layer thickness was approximately evaluated at 0.24 nm [45], so the interlayer regions thickness could be estimated at 0.64 nm.

**Figure 2:**
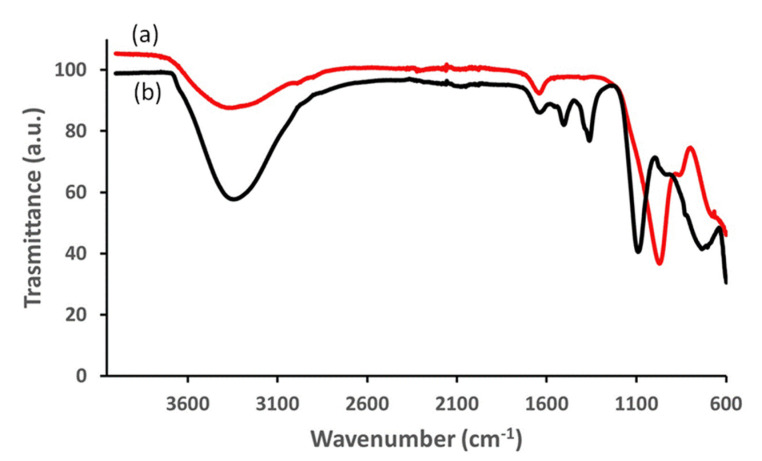
IR spectra for allophane (**a**) and for ZnAl-SO_4_ LDH (**b**).

The IR analysis was performed to check for the presence of undesired ions (e.g., carbonate) in the interlayer of the compounds. The curves of Figure 2a,b show the infrared spectra of allophane and LDH, respectively. In regard to allophane, a large adsorption peak is observable at 3365 cm^−1^, due to the hydrogen bond stretching, confirming the presence of water. The peak at 1640 cm^−1^ is attributable to the H-O-H bending from the crystallization water. The absorption peak at 960 cm^−1^ is typical of the Si-O-Si stretching indicating the presence of Si-O-(Al) bonds. The Si-OH group are responsible for the shoulder at 860 cm^−1^.

In Figure 2b, the infrared spectrum of the LDH reveals the absorption band of the hydrogen bond stretching at 3405 cm^−1^ with a shoulder at higher wavenumber due to the stretching of the hydroxyl bonded with the interlamellar water. At 1630 cm^−1^, the H-O-H bending signal appears. At about 1100 cm^−1^ a deep absorption signal attributable to the sulphate is detectable, while the two small signals at about 1400 cm^−1^ may be due to a few impurities of interlayer carbonate, unavoidable even if the synthesis was carried out in inert atmosphere.

**Figure 3:**
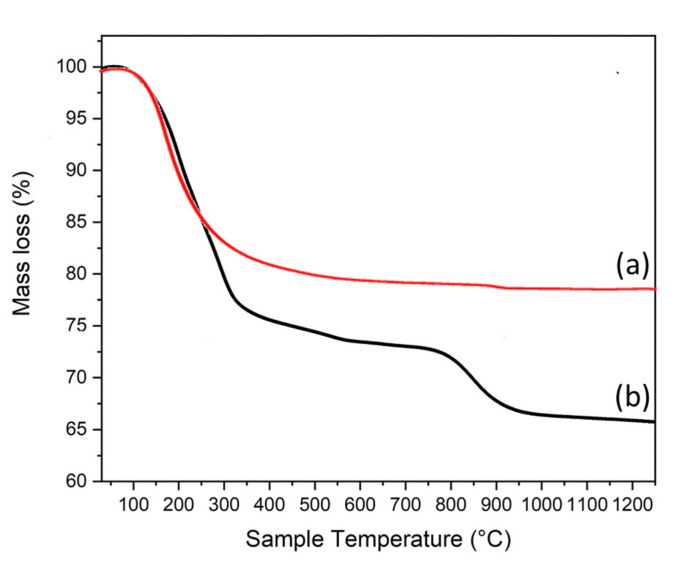
TGA curves for allophane (**a**) and for ZnAl-SO_4_ LDH (**b**).

The thermogravimetric analysis of allophane, visible in curve (a) of Figure 3, shows two mass losses: the first, starting at 80 °C and continuing up to 650 °C, involves a mass decrease of about 20% and it is due to humidity and crystallization water, while the second mass loss, of approximately 0.06%, occurs at about 880 °C. Explaining this last thermal effect is not a simple task, but it could be ascribed to a degradation/dissociation of the substrate, owing to the high temperature attained in the last step of the heating process. In the thermogravimetric pattern for ZnAl-SO_4_ LDH, observable in curve (b) of Figure 3, three mass losses are detectable. The first thermal effect, starting at about 100 °C, is related to a mass decrease of about 8%, due to the humidity, while the second one, occurring in a temperature range of 200–550 °C and leading to a mass decrease of 18%, corresponds to the loss of crystallization water and probably to a release of CO_2_ entrapped in the structure (as seen in the FTIR spectrum). The last thermal effect, starting at about 710 °C, is attributable to SO_2_ gas evolution (7 mass%).

**Figure 4:**
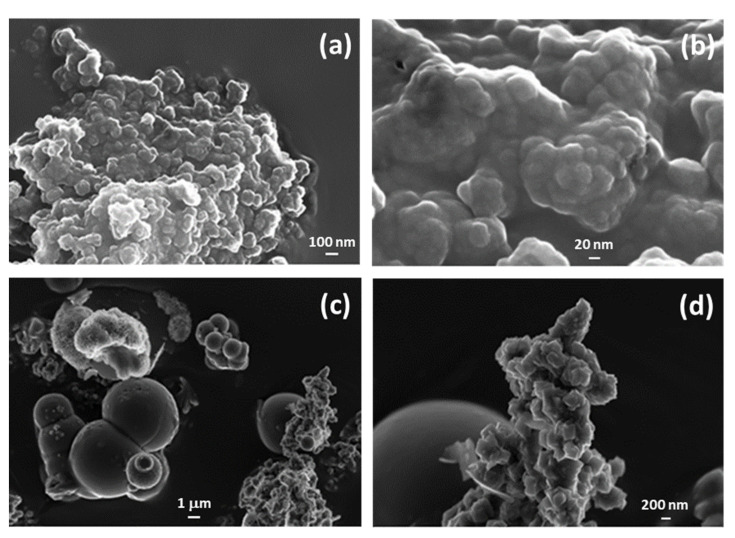
FESEM images for allophane (**a**,**b**) and for ZnAl-SO_4_ LDH (**c**,**d**) at different levels of magnification.

In Figure 4, the microstructure of both sorbents is shown at two different magnifications by FESEM, thus allowing the appreciation of significant details of the structure. In the case of allophane (panels a-b), the primary particles show a pseudo-spherical shape, with diameter in a range 20–50 nm. Xia et al. [46] synthesized allophane following a co-precipitation protocol very similar to the one adopted in the present study. The transmission electron microscopy (TEM) images of the allophane obtained by the authors showed some features with remarkable similarities to the ones observable in Figure 4a,b. They reported a considerable tendency of particle aggregation, with formation of clusters spanning hundreds of nanometers. Analogously, the primary particles had a diameter of approximately 20 nm, in good agreement with the present results of Figure 4b. FESEM analysis of the LDH (Figure 4c,d) reveals that the same compound exhibits different microstructures at different magnification levels, due to a different ratio of nanostructuration, globular and flower-like in shape. These morphologies are typically dependent on the synthesis pathway adopted, whose pH has a key role [47].

### 3.2. Tuning of Sorbent Properties

The adsorption efficiency of the two synthesized materials was tested for different pollutants (cationic and anionic) contained in an industrial wastewater sample, according to a procedure described in previous work [48]. For both solid adsorbents, a weight of 0.5 g of the compound was added to a volume of 100 mL of wastewater, whose pH was kept in a range of 5.0 ± 0.3, by adding dropwise an aqueous solution of 0.5 M NaOH. Additionally, a solid matrix of the same weight, made of 50% allophane and 50% LDH, was tested for comparison in order to ascertain the effects of a mixed composition on the global performances.

The adsorption of metals by allophane at different pH values depends on a pH threshold value corresponding to its point of zero charge (PZC) [36]. This value refers to the conditions needed to obtain a null electrical charge density at the surface. When pH is assumed as a tuning variable, the experimentally determined PZC of allophane is 8.38. For pH values below the PZC, the surface charge of the substrate is positive.

In order to avoid a marked inhibition of cation adsorption, the surface charge of the compound should not be exceedingly positive, thereby favoring the choice of higher pH values. However, for growing pH, mixed hydroxides of various composition may be formed in solution, from which they tend to separate by flocculation or by different precipitation phenomena [49]. Such a process overlaps the adsorption and may interfere with it. A satisfactory trade-off is reached at pH 5.0 ± 0.3 where the synthesized allophane retains its exchange capability [23].

As far as LDH is concerned, low pH values may negatively affect the sorption efficiency of LDH, owing to a growing solubility of the solid matrix for an increasing acidity of the embedding liquid. Unlike allophane, LDH efficiency is not significantly affected by the presence of a PZC threshold value. Experimental tests allowed the conclusion that pH = 6.4 is the smallest pH value providing a LHD stability while preserving its unchanged adsorption capacity.

In the execution of the sorption tests at the equilibrium, the mixtures were left on a rotary shaker for 24 h for all experiments in order to ensure the attainment of the steady-state concentration values of the polluting species, both in the solution and on the sorbent surface. It should be noted that the time gap assumed here is considerably greater than the waiting time generally chosen in the literature for the attainment of the equilibrium condition. Afterwards, the solids were separated from the liquid by centrifugation at 7500 rpm; they were repeatedly washed with water and finally dried in a stove at 50 °C.

The residual wastewater was analyzed before and after the adsorption treatment by ICP-OES. The instrumental detection limit is Cr = 3.2 ppb, Cu = 5.8 ppb and Fe = 10 ppb.

### 3.3. Pollutant Adsorption

The available wastewater sample was analyzed by means of ICP-OES to determine the concentration of the dissolved pollutant metals. The adsorption capacity *q_e_* (mg/g) of each adsorbed polluting species in the solid substrate can be expressed as:

(2) $$q_{e}=\frac{V\left(c_{0}-c_{e}\right)}{m}$$

where *m* is the mass of sorbent used in a volume *V* of liquid hold-up, *c_0_* is the initial concentration (mg/L) of pollutant and *c_e_* is the concentration of pollutant in the liquid phase in equilibrium with the adsorbed species at concentration *q_e_* in the solid phase. The removal rate *r*, namely the percent amount of adsorbed solid with respect to the initial concentration, can be written as:

(3) $$r=\frac{c_{0}-c_{e}}{c_{0}}\cdot 100$$

Freundlich, Langmuir and Temkin models are typically used to relate *q_e_* and *c_e_*, with a marked preference of the first two over the latter in most of the literature devoted to adsorption. In some experimental studies pertaining both to anion and cation adsorption by allophane-like sorbents, the Freundlich model often proved to be more reliable in data-fitting than the Langmuir one [46]. This fact could be ascribed to the intrinsic mechanism underlying the two different schemes, depending on the assumption of a homogeneous/monolayered or a non-homogeneous/multilayered adsorption scheme corresponding to Langmuir or Freundlich models, respectively [50].

As previously mentioned, one of the most important aspects pertaining to the present work stems from the use of real wastewater produced by a metalworking industry, in place of an artificial dispersion of selected ions simulating a polluting emission, which is the typically adopted protocol by the literature related to this topic. The concentration values of the different ionic species initially present in the wastewater were 0.57 ppm Cu(II), 0.028 ppm Fe(III) and 102 ppm Cr(VI). The values for Cu(II) and Cr(VI) were higher than allowed by the legal limits set down by Italian law both for superficial and drainage water [51]. After the batch equilibration procedure, the pH value of the residual wastewater did not change significantly. The results are reported in Table 1.

In case of Cr(VI) adsorption, the joint use of Equation (2) and the data of Table 1 allow us to calculate that the values of *q_e_* for allophane and ZnAl-SO_4_ LDH are 3.2 and 10.8 mg/g, respectively.

Pranoto et al. [52] synthesized an allophane-like sorbent with a sol-gel technique using tetraethyl orthosilicate and aluminum nitrate as precursors in various proportions, aiming to obtain several sorbents with different Al/Si ratios. Their estimated Freundlich parameters allowed us to calculate that, for *c_e_* = 86 mg/L of Cr(VI) from Table 1, the corresponding *q_e_* is equal to 1.64. This value is significantly lower than the one (*q_e_* = 3.2) experimentally obtained in the present study, probably owing to the positive effects related to the preparation technique adopted here. In another study, Babel and Opiso [53] investigated the properties of two volcanic ash soils as sorbents for Cr(VI) contained in synthetic wastewater. Allophane and imogolite in amorphous state were the main constituents of their solid substrate, whose kinetics in Cr(VI) capture were particularly fast, needing a time not exceeding 3 h in all experimental tests. From their data-fitting concerning the Freundlich isotherm, the calculated *q_e_* for c*_e_* = 86 mg/L were 3.08 and 3.49 mg/g for the two different sorbents, proving that our results for Cr(VI) adsorption on ZnAl-SO_4_ allophane are consistent with their findings.

Hu et al. [54] adopted a mechanochemical method as an alternative to wet chemical processes to prepare MgAl-LDH and a LDH nanocomposite with expanded graphite, and they obtained powders whose physical properties differed from one another according to the ball-milling speed and time. From sorption tests at equilibrium carried out for LDH and its graphite composite, they obtained the values of Freundlich, Langmuir and Temkin parameters by data regression. For comparison, one can use their values of Freundlich parameters to calculate the value of *q_e_* corresponding to the equilibrium value *c_e_* = 50.6 reported in Table 1 for our ZnAl-SO_4_ LDH in the case of Cr(VI) adsorption, thus obtaining *q_e_* = 11.59 and *q_e_* = 11.50. Both values are very close to the one (*q_e_* = 10.8) pertaining to the ZnAl-SO_4_ LDH sorbent investigated here.

It is important to point out that the present study is not instrumental in finding the best performances in terms of Cu(II) and Fe(III) adsorption. Instead, the abatement of Cr(VI) represents the main target of these experimental tests, owing to the well-known negative impacts of Cr(VI) on the environment and human health. In this context, Cr(VI) may have toxic and mutagenic effects by far higher than the one exerted by other cations considered here. In the results reported in Table 1, some basic aspects should be remarked upon, namely:

- In terms of adsorption capacity *q_e_*, allophane, ZnAl-SO_4_ LDH and their combination offer a greater affinity towards Cr(VI) anions rather than cations such as Cu(II) and Fe(III). However, reflecting on removal rate rather than adsorption capacity, one can observe a symmetrical behavior between allophane and ZnAl-SO_4_ LDH. In fact, allophane showed a satisfactory efficiency for both cations, while it is barely performant for chromate anion. On the other hand, LDH is a better absorbent for chromate anion than for copper cation. Actually, with the data at our disposal, it is difficult to draw definitive conclusions about the performance for Fe(III) owing to its very low initial concentration in wastewater.
- A presence of cations such as Cu(II) and Fe(III), almost ubiquitous in real metalworking wastewaters, does not interfere with the adsorption of Cr(VI), here dissolved in anionic form. Even despite the presence of these host cations, a 50% combination of allophane and LDH gives a value of *q_e_* for Cr(VI) adsorption very close to the maximum between the two components.
- Notwithstanding the small values of *q_e_* for Cu(II) and Fe(III) adsorption obtainable from the data of Table 1, a synergistic effect in terms of Cu(II) removal rate is observable for a 50% allophane/LDH combination.

The last point deserves some further theoretical insights aiming to quantify the synergistic effects set out therein. Recently, some authors analyzed the effects of multiple superposed phenomena on pollutant degradation kinetics in aqueous medium by sonocatalysis, photocatalysis and combined sonophotocatalysis on MOFs [55] and on bimetallic oxides [56]. It is interesting to extend their effective approach, concerning reaction kinetics, to a context related to thermodynamics of sorption equilibria, which is pertinent to the present study.

To this purpose, the synergy index *ϕ* is here defined as follows:

(4) $$\phi =\frac{q_{e}(allophane+LDH)}{\omega_{A}q_{e}(allophane)+\omega_{LDH}q_{e}(LDH)}$$

where:

$m_{TOT}$, $m_{A}$ and $m_{LDH}$ are the mass of total solid, allophane and ZnAl-SO_4_ LDH in the hold-up, respectively;

$\omega_{A}=\frac{m_{A}}{m_{TOT}}$ and $\omega_{LDH}=\frac{m_{LDH}}{m_{TOT}}$ are the allophane and ZnAl-SO_4_ LDH mass fractions, respectively.

If allophane and ZnAl-SO_4_ LDH would act separately in the adsorption process, namely independently of one another, the total adsorbed solid from the liquid phase at equilibrium would be:

(5) $$m_{TOT}q_{e}(allophane+LDH)=m_{A}q_{e}(allophane)+m_{LDH}q_{e}(LDH)$$

And finally, dividing both sides of Equation (5) by $m_{TOT}$, one obtains:

(6) $$q_{e}(allophane+LDH)=\omega_{A}q_{e}(allophane)+\omega_{LDH}q_{e}(LDH)$$

with a resulting synergy index *ϕ* = 1.

In case of a synergy between the two sorbents, it is expected *ϕ* > 1.

Taking into account Equation (2) and the data reported in Table 1 for Cu(II) adsorption, one can easily calculate *ϕ* (Cu) = 1.545, thus attaining a quantitative evaluation of the synergy effect between allophane and ZnAl-SO_4_ LDH for Cu(II) adsorption. It must be stressed that the corresponding value *ϕ* (Fe) for Fe(III) adsorption could not be calculated, owing to an equilibrium concentration of Fe(III) in the wastewater below the detection limit of the instrument.

In analogy with the approach adopted by Hassandoost et al. [56], a degradation turnover *dTON* can be defined as follows:

(7) $$dTON=\frac{C_{0}-C_{e}(t)}{t\cdot m_{TOT}}$$

where *C_0_* is the initial molar concentration of a generic pollutant and *C_e_(t)* is its final molar concentration at time *t*. Using the data reported in Table 1 for Cr(VI) adsorption, one obtains dTON(Cr) values equal to 2.56, 8.24 and 7.85 μmol h^−1^g^−1^ for allophane, LDH and allophane + LDH mixture, respectively. In case of Cu(II) adsorption, one obtains dTON(Cu) values equal to 0.065, 0.02 and 0.067 μmol h^−1^g^−1^ for allophane, LDH and allophane + LDH mixture, respectively. Each dTON value for Cu adsorption is considerably smaller than its corresponding for Cr adsorption, owing to a great difference between the initial molar concentration *C_0_* of the two cations.

As for the chromate adsorption mechanism by LDH, the results seem to strengthen the hypothesis that chromate anion replaces the sulphate anion, owing to their similarity in dimension and charge. In fact, the cell parameters of the LDH chromate-substituted are the following: a = 0.3045(2) nm, c = 2.6537(5) nm, V = 0.2131 nm^3^. These values are very similar with those typical of the pristine LDH.

**Figure 5:**
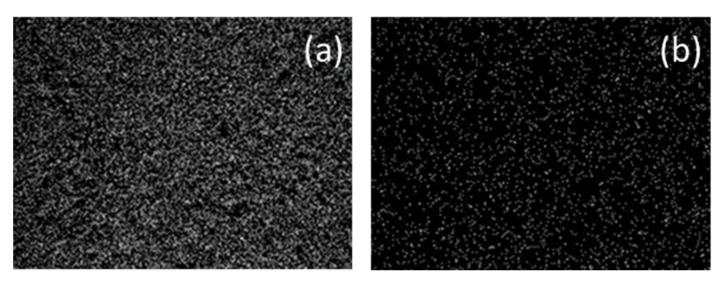
SEM analysis of ZnAl-SO_4_ LDH after adsorption. The white colored areas refer to Al(III) and Cr(III) in panels (**a**,**b**), respectively.

Figure 5 shows a SEM analysis of the LDH after chromate adsorption. The white dots in panel (a) suggest an approximately uniform distribution of Al(III) in the substrate related to its pristine characteristics, while they refer to Cr(VI) in panel (b).

The concentration data reported in Table 2 are the results of an average of five different energy-dispersive x-ray spectroscopy (EDS) analyses carried out on the specimen which Figure 5 refers to. The concentration of other cations, taking into account their dilution ratio with respect to the anion Cr(VI) in the wastewater, was below the detection limit of the instrument.

**Figure 6:**
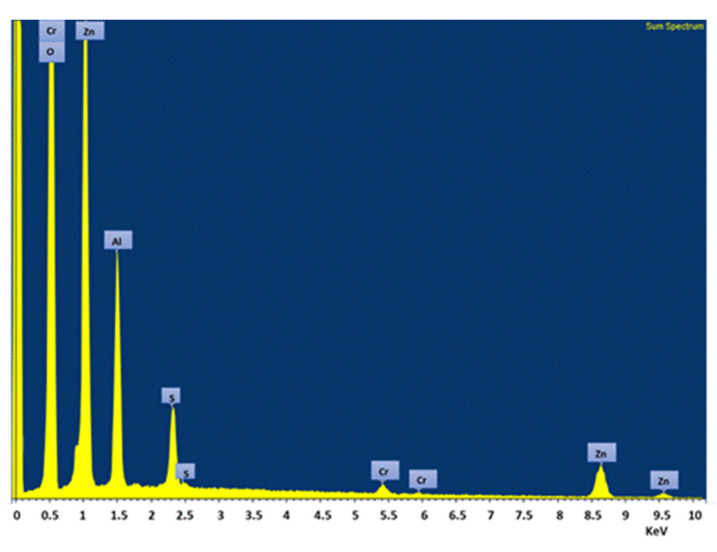
EDS spectrum of elements present in ZnAl-SO_4_ LDH substrate after adsorption of ions from wastewater.

In Figure 6, the EDS spectrum of the elements contained in the above mentioned LDH substrate after adsorption is reported. The leftmost peak is a spurious signal of the instrument.

An in-depth speculation about the reasons which explain the differences of LDH affinities for the two cations is beyond the scope of the present work and it will be the object of further investigations in another study. The adsorption mechanism for copper seems to be attributable to an exchange between the copper and zinc divalent cations, allowed by the similar ionic radius values of the two elements in octahedral coordination (72 and 74 pm, respectively), even if other sorption mechanism (e.g., bond on the OH- functional groups, complexation) could not be completely excluded [48].

From a strictly applicational point of view, the treatment of real wastewater with the two sorbents mixed together leads to a decontamination both from the cations and from the anion. Interestingly, neither cation nor anion adsorption is negatively affected by a mix of both sorbents in the same solid phase.

## 4. Conclusions

Two different adsorbent compounds were synthesized and characterized, before being tested for remediation purposes on industrial wastewater produced by a metalworking plant. This study refers to experimental tests carried out on a non-simulated wastewater and, in this respect, it represents an atypical approach compared to traditional schemes proposed in literature where artificial wastewater is generally employed. The most important findings can be summarized in the following points:

- The synthesis is simple, cost-effective and sustainable, both for allophane and for ZnAl-SO_4_ LDH: according to inherent safety guideword “substitution”, no reagents hazardous for health or environment were employed.
- In a context pertaining to the adsorption capacity, allophane, ZnAl-SO_4_ LDH and their combination proved to be efficient in the adsorption of Cr(VI).
- The removal rate of each sorbent species showed different trends according to their physicochemical properties, leading to different scenarios in wastewater decontamination. Being an anionic clay, allophane proved to be more efficient for cation extraction, while LDH showed a greater affinity for the anion which can be exchanged with the one originally present in the interlayer of the pristine substrate.
- The synergy effect between allophane and ZnAl-SO_4_ LDH for Cu(II) adsorption has been quantified according to the synergy index approach.
- The affinity typical of ZnAl-SO_4_ LDH for the chromate anion, though attaining the highest value with respect to the other sorbent and mixture tested here, was lower than other LDH-type sorbents considered in another work [48]. Nevertheless, its application in wastewater remediation can be interesting if the release of sulphate instead of other anions (eg. carbonate, nitrate) from the LDH during the reaction is preferred.
- While the LDH exchange mechanism for the anions occurs in the interlayer, a cationic substitution in the brucite layer can be hypothesized if the ionic rays of the new and leaving species are similar. Regarding iron adsorption, the characterization analysis did not provide precise information on where it may have taken place. It is possible that iron has been removed via interaction with the functional groups on the surface of the mineral.
- A future development of the present study will be devoted to realizing the highest-performing experimental setup in order to optimize the remediation purposes by combining the solid sorbents here proposed in different proportions.

## Figures and Tables

**Table 1:** Equilibrium concentration value and removal rate of ionic species in the presence of a constant mass (5 g/L) of different sorbents. The data are averaged on triplicate samples.

Wastewater Treatment	Metals					
	Cr(VI)		Cu(II)		Fe(III)	
	Concentration (ppm)	Removal Rate r (%)	Concentration (ppm)	Removal Rate r (%)	Concentration (ppm)	Removal Rate r (%)
Untreated	102	/	0.57	/	0.03	/
Allophane	86	15.7	0.07	87.7	0.01	66.6
ZnAl-SO_4_ LDH	50.6	50.4	0.41	28.0	<d.l.	~100
Allophane + LDH	53.0	48	0.06	89.5	<d.l.	~100

**Table 2:** Percent mass distribution of the main elements present in the sample considered in Figure 5.

Element	Mass % (Average Value)
Al	13.39
S	5.29
Cr	0.44
Zn	45.14
O	35.74
Total	100.00

## Data Availability

Not applicable.
